# Laser-processed antiadhesive bionic combs for handling nanofibers inspired by nanostructures on the legs of cribellate spiders

**DOI:** 10.3762/bjnano.13.105

**Published:** 2022-11-07

**Authors:** Sebastian Lifka, Kristóf Harsányi, Erich Baumgartner, Lukas Pichler, Dariya Baiko, Karsten Wasmuth, Johannes Heitz, Marco Meyer, Anna-Christin Joel, Jörn Bonse, Werner Baumgartner

**Affiliations:** 1 Institute of Biomedical Mechatronics, Johannes Kepler University Linz, Altenberger Straße 69, 4040 Linz, Austria; 2 Bundesanstalt für Materialforschung und -prüfung (BAM), Unter den Eichen 87, 12205 Berlin, Germany; 3 Institute of Applied Physics, Johannes Kepler University Linz, Altenberger Straße 69, 4040 Linz, Austria; 4 Institute of Biology II (Zoology), RWTH Aachen University, Worringerweg 3, 52074 Aachen, Germany

**Keywords:** biomimetics, electrospinning, laser-induced periodic surface structures (LIPSS), nanofibers, nanostructures

## Abstract

Nanofibers are drawing the attention of engineers and scientists because their large surface-to-volume ratio is favorable for applications in medicine, filter technology, textile industry, lithium-air batteries, and optical sensors. However, when transferring nanofibers to a technical product in the form of a random network of fibers, referred to as nonwoven fabric, the stickiness of the freshly produced and thus fragile nanofiber nonwoven remains a problem. This is mainly because nanofibers strongly adhere to any surface because of van der Waals forces. In nature, there are animals that are actually able to efficiently produce, process, and handle nanofibers, namely cribellate spiders. For that, the spiders use the calamistrum, a comb-like structure of modified setae on the metatarsus of the hindmost (fourth) legs, to which the 10–30 nm thick silk nanofibers do not stick due to a special fingerprint-like surface nanostructure. In this work, we present a theoretical model of the interaction of linear nanofibers with a sinusoidally corrugated surface. This model allows for a prediction of the adhesive interaction and, thus, the design of a suitable surface structure to prevent sticking of an artificially nonwoven of nanofibers. According to the theoretical prediction, a technical analogon of the nanoripples was produced by ultrashort pulse laser processing on different technically relevant metal surfaces in the form of so-called laser-induced periodic surface structures (LIPSS). Subsequently, by means of a newly established peel-off test, the adhesion of an electrospun polyamide fiber-based nonwoven was quantified on such LIPSS-covered aluminium alloy, steel, and titanium alloy samples, as well as on polished (flat) control samples as reference and, additionally, on samples with randomly rough surfaces. The latter revealed that the adhesion of electrospun nanofiber nonwoven is significantly lowered on the nanostructured surfaces compared with the polished surfaces.

## Introduction

Nanofibers have a diameter of approximately 10 to 800 nm, whereas their length is much greater than their diameter, which is why the term fiber or thread is used. These fibers are constantly drawing the attention of engineers because their surface-to-volume ratio is favorable for applications in medicine, filter technology, textile industry, lithium-air batteries, and optical sensors [1–7].

The inherently small scale makes production as well as further processing of nanofibers challenging [8]. For the technical production of artificial nanofibers, different methods such as electrospinning [1–2,6–8] or microfluidic spinning [4] have been established. Despite a lot of effort to facilitate the production and handling of nanofibers [3–7], the stickiness of freshly produced and, thus, fragile nanofiber nonwoven mats remains a problem. This is mainly because nanofibers strongly adhere to any surface due to van der Waals forces [9]. For a cylindrical fiber with radius *R* interacting with the plane surface of a semi-infinite body, the energy per unit length due to van der Waals interaction is given as [9]:


[1] $$\mu =-\sqrt{2R}\cdot \frac{A_{\text{H}}}{24d^{3/2}},$$

with the Hamaker constant *A*_H_, which is according to [9]:


[2] $$A_{\text{H}}=\pi^{2}\cdot \rho_{1}\cdot \rho_{2}\cdot c.$$

Here, the mass densities (ρ_1_ and ρ_2_) of the interacting bodies and the London coefficient *c*, which describes the particle–particle interaction, are multiplied.

The van der Waals energy *U*_vdW_ of the fiber obtained due to the interaction is the integral of the above interaction function µ over the entire fiber length *l*, which can depend on the position along the fiber:


[3] $$U_{\text{vdW}}=\int_{l}\mu \left(l\right)\text{d}l.$$

This formulation could be interpreted in such a way that if the radius of the fiber decreases, the van der Waals force also decreases and can, therefore, be neglected if *R* is sufficiently small. However, this is not always the case for these reasons: (1) Due to the smaller radius, the fiber also becomes softer. In consequence, the easier deflection can increase the contact area, resulting in larger forces. The van der Waals force is proportional to the root of the radius, μ ∼ √*R*, and the materials stiffness, expressed by the area moment of inertia *J*, is proportional to the fourth power of the radius, *J* ∼ *R*^4^. Hence, the fiber gains more contact area much faster than the force decreases. (2) With a smaller radius, more fibers can attach simultaneously to the surface, which leads to a further increase in the total interacting surface area.

**Figure 1:**
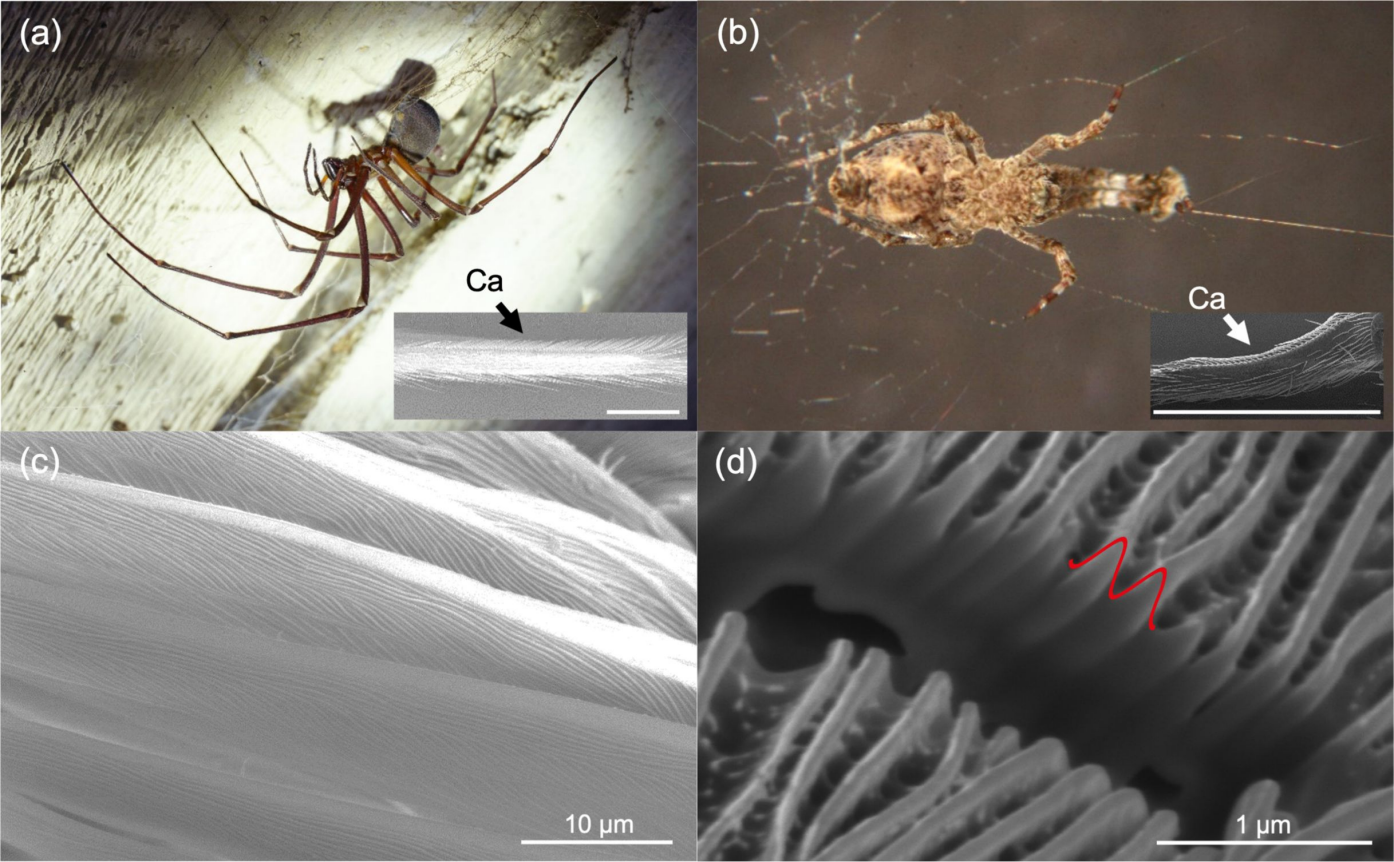
Images of four distantly related and differently sized cribellate spiders with same-sized nanoripples covering their calamistrum (antiadhesive comb to handle nanofibers). (a) Photography of the endemic and last species of an old Gondwanan lineage, the Tasmanian cave spider *Hickmania troglodytes* (body size of up to 2 cm [15] and a leg span up to 18 cm [16]). (b) Photography of the cosmopolitan feather-legged lace weaver *Uloborus plumipes* (body size up to 0.6 cm [17]). (c) Scanning electron micrograph of the calamistrum of *Jamberoo johnnoblei* (body size up to 0.8 cm [18]). (d) FIB-cut high resolution SEM image through the nanoripples on the calamistrum of the lace-webbed spider *Amaurobius similis* (body size up to 1.2 cm [19]), highlighting in red the abstracted sinusoidal surface corrugation as cross-sectional profile of the nanoripples. The insets in (a) and (b) provide SEM micrographs of the calamistra (Ca) of the respective spiders, with scale bars of 0.8 mm length. Data is presented in Table S1 of Supporting Information File 1.

Though technical nanofiber handling and processing is limited today, in nature, there are animals that are actually able to efficiently produce, process, and handle nanofibers, namely cribellate spiders [10–11]. Their capture thread consists of one or two axial fibers as “construction elements” surrounded by a wool of nanofibers. This wool is used to capture prey, deploying van der Waals forces and additionally embedding the fibers into the viscous waxy layer of the insects’ cuticle [12–13]. One thread typically consists of 5000 to 30000 single fibers with a thickness of 10–30 nm. In the spinning process, the spiders extract this silk from the cribellum (spinning plate) and process it by “combing” the fibers to form a puffy structure surrounding the axial fibers [10]. To process the fibers, the spiders use the calamistrum, a comb-like structure of modified setae on the metatarsus of the hindmost (fourth) legs [10] (Figure 1). The 10–30 nm thick silk nanofibers do not stick to the calamistrum due to a special fingerprint-like nanostructure. This was characterized recently [14] for the calamistrum of *Uloborus plumipes* (commonly named feather-legged lace weaver or the garden center spider). Its fingerprint-like outmost surface structure has an approximately sinusoidal cross section with a periodicity of 200–300 nm and a height (amplitude) of approx. 200 nm. During the combing process, the nanofibers are pulled orthogonally over these nanoripples. It was shown that the nanostructure on the calamistrum in fact reduces adhesion of native spider silk and that this reduced adhesion can be mimicked by artificially structured polymer foils [14]. In order to technically integrate these antiadhesive structures, the structures have to be adapted as typical technical nanofibers differ quite significantly from spider silk fibers regarding diameter and material properties (e.g., Young’s modulus).

In this work, we present a theoretical model of the interaction of nanofibers with a sinusoidal surface based on an energy approach. This model allows for a prediction of the adhesive interaction and, thus, the design of a suitable surface structure to prevent sticking of an artificially nonwoven of nanofibers. Similar to the description of the Lotus effect [20–23], where the wettability of the hierarchical surface structure of the lotus leaf can be described with an energy approach related to the surface free energy of the fluid, we use an energy approach related to the bending energy of the nanofibers to describe the fiber adhesion on structured surfaces. An important point is that the theory presented here deals with cylindrical fibers that come into contact with the surface structure, in contrast to earlier published works where solids with plane surfaces were assumed [24–28]. This is an important difference, which has to be considered when modelling the interaction of nanofibers with, in this case, a sinusoidal surface.

Laser-induced periodic surface structures (LIPSS) [29] represent a technical analogon of the nanoripples found on the calamistrum of the spider. They were produced by ultrashort pulse laser processing on different technically relevant metal surfaces (aluminium alloy, steel, and titanium alloy) according to theoretical predictions. Subsequently, the adhesion of electrospun polyamide fibers was quantified on these structures as well as on polished (flat) control samples as reference. In addition, different randomly rough surfaces were investigated and compared to the LIPSS. This bioinspired laser-based surface functionalization paves a new way for technologically improving the production of tools for handling of artificial nanofibers. This can facilitate and optimize the production of, for example, filter materials and nanofabrics.

## Results and Discussion

### Theoretical modelling

**Figure 2:**
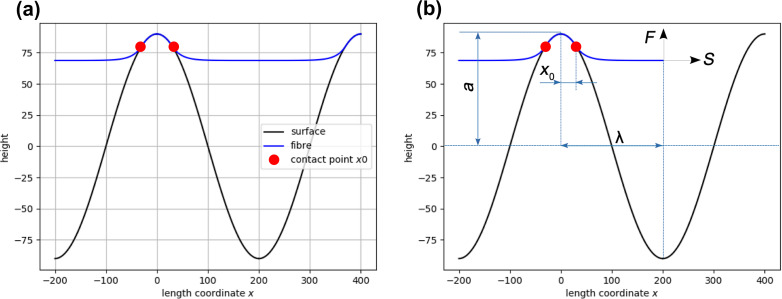
Principle geometry of the interaction of a nanofiber with a periodic sinusoidal surface topography (a) and as single length unit when cut free (b). The periodic surface structure is drawn in black, the fiber is drawn in blue. Surface and fiber are mathematically modelled by two functions, *f*(*x*) and *w*(*x*), respectively. The cross section of the surface is sinusoidal with a period of 2λ and an amplitude of *a*. A fiber on top of the surface is deflected partially due to van der Waals interactions. The point of detachment *x*_0_ is used as a parameter to characterize the system. A longitudinal force *S* can stretch the fiber. For theoretical modelling, a single unit is cut free (b) and the action of the fiber at *x* > λ is replaced by the yet unknown vertical force *F*. This is the force required to reach a value of zero for the slope of the fiber at *x* = λ.

The general model is depicted in Figure 2. The course of the fiber is divided into two areas along the length coordinate *x*. These areas are (1) the contact area, that is, the area between the contact points (marked by red full circles in Figure 2), where the fiber adheres to the surface, and (2) the sagging area, where the fiber has no contact to the surface. The point of detachment of the fiber from the surface is defined as the detachment point *x*_0_. This point can parametrize the curve and is initially assumed to be known. The fiber is assumed to have a certain bending stiffness and, thus, it can be modelled according to the linear elastic (Hookean) beam theory (Euler–Bernoulli beam theory). Thus, we have to solve the ordinary differential equation (ODE) [30]


[4] $$w^{\prime \prime }\left(x\right)=-\frac{M}{EJ},$$

with *w* describing the deflection from the neutral position, *M* denoting the bending moment, *E* describing the Young’s elastic modulus, and *J* denoting the second moment of area (moment of inertia of plane area).

In order to solve the above ODE, we need to know the bending moment and adequate boundary conditions. According to the model depicted in Figure 2 we assume to precisely know the position of the point of detachment *x*_0_. At this location the two boundary conditions are


[5] $$\begin{array}{c} w\left(x_{0}\right)=f\left(x_{0}\right)\text{ and}\\ w^{\prime }\left(x_{0}\right)=f^{\prime }\left(x_{0}\right), \end{array}$$

that is, the mathematical curve describing the fiber *w*(*x*) touches the curve describing the surface profile *f*(*x*). To obtain the bending moment, we cut the fiber virtually at position λ, that is, in the middle of one period of the periodic surface structure. We know that a longitudinal force *S* is applied to the fiber in natural as well as in technical nanofiber production, and we assume a force *F* acting vertical at the position of the cut, λ, to bring the system back into equilibrium. This force is used to make the slope *w*′(λ) assume a value of zero, that is, there is a horizontal tangent and, thus, *w*′(x) is continuous and continuously differentiable at position λ. Hence, the ODE in Equation 4 can be solved by generally assuming a force *F* and calculate *F* according to the requirement that *w*′(λ) = 0.

The general solution of Equation 4 can be written as


[6] $$\begin{array}{c} w\left(x\right)=\frac{F\cdot \lambda }{S}-\frac{F\cdot x}{S}+w\left(\lambda \right)+C_{1}\cdot \exp\left[\sqrt{\frac{S}{EJ}}\cdot \left(x-\lambda \right)\right]\\ +C_{2}\cdot \exp^{-1}\left[\sqrt{\frac{S}{EJ}}\cdot \left(x-\lambda \right)\right]. \end{array}$$

For the following, this can be simplified as, at position *x* = λ, Equation 6 becomes


[7] $$w\left(\lambda \right)=C_{1}+C_{2}+w\left(\lambda \right).$$

Thus, we obtain directly


[8]

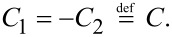



After substituting Equation 8 into Equation 6 and performing a derivation with respect to *x*, we obtain *w*′(*x*) as


[9] $$\begin{array}{c} w^{\prime }\left(x\right)=-\frac{F}{S}+C\cdot \sqrt{\frac{S}{EJ}}\\ \cdot \left\{\exp\left[\sqrt{\frac{S}{EJ}}\cdot \left(x-\lambda \right)\right]+\exp^{-1}\left[\sqrt{\frac{S}{EJ}}\cdot \left(x-\lambda \right)\right]\right\}. \end{array}$$

In order to obtain *w*′(λ) = 0, we can insert *x =* λ in Equation 9, yielding


[10] $$w^{\prime }\left(\lambda \right)=-\frac{F}{S}+C\cdot \sqrt{\frac{S}{EJ}}\cdot 2=0\Rightarrow F=2S\cdot C\cdot \sqrt{\frac{S}{EJ}}.$$

After inserting Equation 10 into Equation 6 and using the definition of the hyperbolic sine, we obtain


[11] $$w\left(x\right)=2C\cdot \left[\sinh\left(\sqrt{\frac{S}{EJ}}\cdot \left(x-\lambda \right)\right)\cdot \left(x-\lambda \right)\right]+w\left(\lambda \right).$$

Now, to fulfil the condition in the second part of Equation 5, we have to differentiate Equation 11 and solve the equation so that *w*′(*x* = *x*_0_) = *f*′(*x* = *x*_0_).


[12]

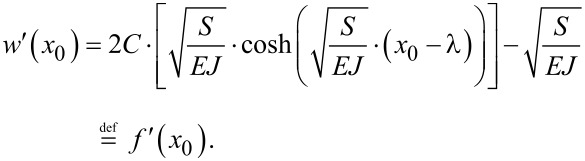



This directly yields


[13] $$C=\frac{f^{\prime }\left(x_{0}\right)}{2\cdot \sqrt{\frac{S}{EJ}}\cdot \cosh\left(\sqrt{\frac{S}{EJ}}\left(x_{0}-\lambda \right)\right)}.$$

Finally, in order to fulfil the boundary conditions *w*(*x* = *x*_0_) = *f*(*x* = *x*_0_), one has to calculate *w*(λ), which can be obtained from


[14] $$w\left(\lambda \right)=f\left(x_{0}\right)-C\cdot \left[\sinh\left(\sqrt{\frac{S}{EJ}}\cdot \left(x_{0}-\lambda \right)\right)-\sqrt{\frac{S}{EJ}}\cdot \left(x_{0}-\lambda \right)\right].$$

Taken together, if the topography function of the surface structure *f*(*x*) is given, the bending line, which only depends on the position of the contact point *x*_0_, is given to be


[15] $$\begin{array}{c} w\left(x\right)=2C\cdot \left[\sinh\left(\sqrt{\frac{S}{EJ}}\cdot \left(x-\lambda \right)\right)\cdot \left(x-\lambda \right)\right]+w\left(\lambda \right)\\ \text{with }C=\frac{f^{\prime }\left(x_{0}\right)}{2\sqrt{\frac{S}{EJ}}\cdot \cosh\left(\sqrt{\frac{S}{EJ}}\left(x_{0}-\lambda \right)\right)}\\ \text{and }w\left(\lambda \right)=f\left(x_{0}\right)-C\cdot \left[\sinh\left(\sqrt{\frac{S}{EJ}}\cdot \left(x_{0}-\lambda \right)\right)-\sqrt{\frac{S}{EJ}}\cdot \left(x_{0}-\lambda \right)\right]. \end{array}$$

A special case should be considered separately, namely *S* = 0, that is, if no longitudinal force is applied and the fiber can freely form contact with the surface. Instead of reformulating the above result using L'Hôpital's rule, we could simply solve the initial differential Equation 4 with *S* = 0, which directly leads to


[16] $$\begin{array}{c} w(x)=f(x_{0})+\frac{F}{EJ}\cdot \left(\frac{x_{0}^{3}}{3}-\frac{x\cdot x_{0}^{2}}{2}-\frac{\lambda \cdot x_{0}^{2}}{2}+\frac{\lambda \cdot x\cdot x_{0}}{1}-\frac{\lambda \cdot x^{2}}{2}+\frac{x^{3}}{6}\right)\\ +f'(x_{0})\cdot (x-x_{0}). \end{array}$$

When, now, *F* is adjusted so that *w*′(λ) = 0, we obtain for this special case without longitudinal force


[17] $$w\left(x\right)=f\left(x_{0}\right)-f^{\prime }\left(x_{0}\right)\cdot \frac{x_{0}^{2}+\left(3\lambda -x\right)\cdot x_{0}+2x^{2}-3\lambda \cdot x}{3\left(x_{0}+x+2\lambda \right)}.$$

Now, in any case, the position *x_0_* of the contact point remains to be determined. For this, we need to find the energy minimum of the system with respect to *x*_0_, which would correspond to the position where, in equilibrium, the fiber detaches from the surface structure. There are three energetic contributions to the energy of the whole system that depend on *x*_0_:

(i) The bending energy of a fiber in contact with the surface, that is, the elastic energy stored in the fiber in the region of contact from *x* = 0…*x*_0_, which can be calculated for a linear elastic beam as


[18] $$E_{1}\left(x_{0}\right)=\frac{EJ}{2}\cdot \int_{0}^{x_{0}}w^{\prime \prime }{\left(x\right)}^{2}\text{d}x=\frac{EJ}{2}\int_{0}^{x_{0}}f^{\prime \prime }{\left(x\right)}^{2}\text{d}x.$$

(ii) The bending energy of the free fiber, that is, in the region of no contact from *x* = *x*_0_…λ, which follows to be


[19] $$E_{2}\left(x_{0}\right)=\frac{EJ}{2}\cdot \int_{x_{0}}^{\lambda }w^{\prime \prime }{\left(x\right)}^{2}\text{d}x.$$

(iii) Finally, the van der Waals energy which, according to Equation 3, follows to be


[20] $$E_{3}\left(x_{0}\right)=-\int_{0}^{x_{0}}\mu \left(x\right)\cdot \sqrt{1+w^{\prime }{\left(x\right)}^{2}}\text{d}x.$$

It must be emphasized that, in principle, the above term can also account for other (additional) attractive energies such as electrostatic interactions. All these can be summarized in the term µ(*x*) and can, therefore, depend on the position and, thereby, on the distance of fiber and surface (*w*(*x*) − *f*(*x*)).

However, only the van der Waals energy *E*_3_ exhibits a negative sign and, thus, decreases the total energy stored in the system, leading to more deflection and, thus, to more contact of the fiber with the surface. In contrast, the elastic energies *E*_1_ and *E*_2_ increase the total energy,


[21] $$E_{\text{tot}}\left(x_{0}\right)=E_{1}\left(x_{0}\right)+E_{2}\left(x_{0}\right)+E_{3}\left(x_{0}\right).$$

In order to find the fiber detachment point *x*_0_ in equilibrium, one needs to solve the optimization problem


[22] $$\frac{\partial E_{\text{tot}}}{\partial x_{0}}=0.$$

A general analytical solution to this optimization problem for any surface, that is, for any *f*(*x*), cannot be found. However, numerical solutions are easily possible and, for some cases, even approximate analytical solutions are available. Let us assume the surface to be described by a cosine function (that is obviuosly identical with a laterally shifted sine function), that is,


[23] $$f\left(x\right)=a\cdot \cos\left(\frac{\pi \cdot x}{\lambda }\right).$$

It has to be emphasized that, if the periodic modulation on the surface has a sinusoidal cross section but the fiber is not orthogonally oriented to these ridges, the cross section under an angle is still represented by a cosine function with same amplitude *a* but with a different characteristic length λ. Thus, the formal description derived here is still valid.

Due to the high exponent of the dependence of van der Waals energy and distance we can approximate the system and assume that we have a constant µ = µ_vdW_ from *x* = 0…*x*_0_ and µ = 0 from *x = x*_0_…λ. For this, we obtain the energy contributions in the case of a finite longitudinal force *S* as given below in Equation 24.


[24]

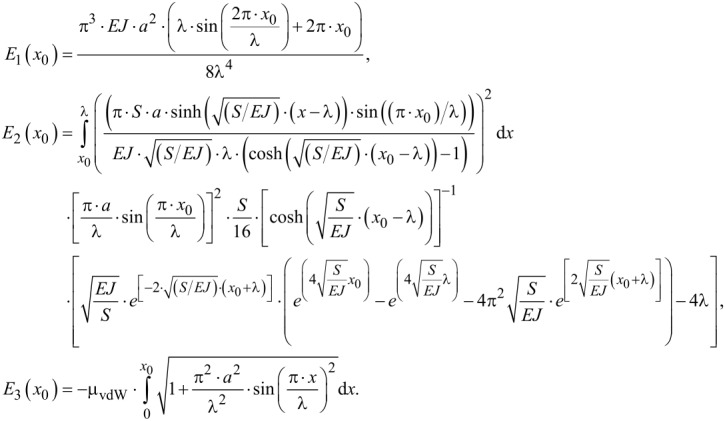



The integral for *E*_3_ can be solved using complex incomplete elliptic integrals of the second kind. These can be calculated numerically. The system tends to the state of minimal total energy, *E*_1_ + *E*_2_ + *E*_3_ → min.

In principle, there are three possible states: In state A, it requires more energy to bend the fiber to adapt to the surface than can be gained due to van der Waals interaction. A straight fiber touches only the tips of the surface. In state B, it is energetically favorable to deflect the fiber in order to obtain the interaction energy, but not all to the bottom of the sinusoidal surface topography. Thus, a clear total energy minimum exists. Finally, in state C, bending requires less energy than can be gained by the van der Waals interaction. Thus, the fiber adapts completely to the shape of the surface.

**Figure 3:**
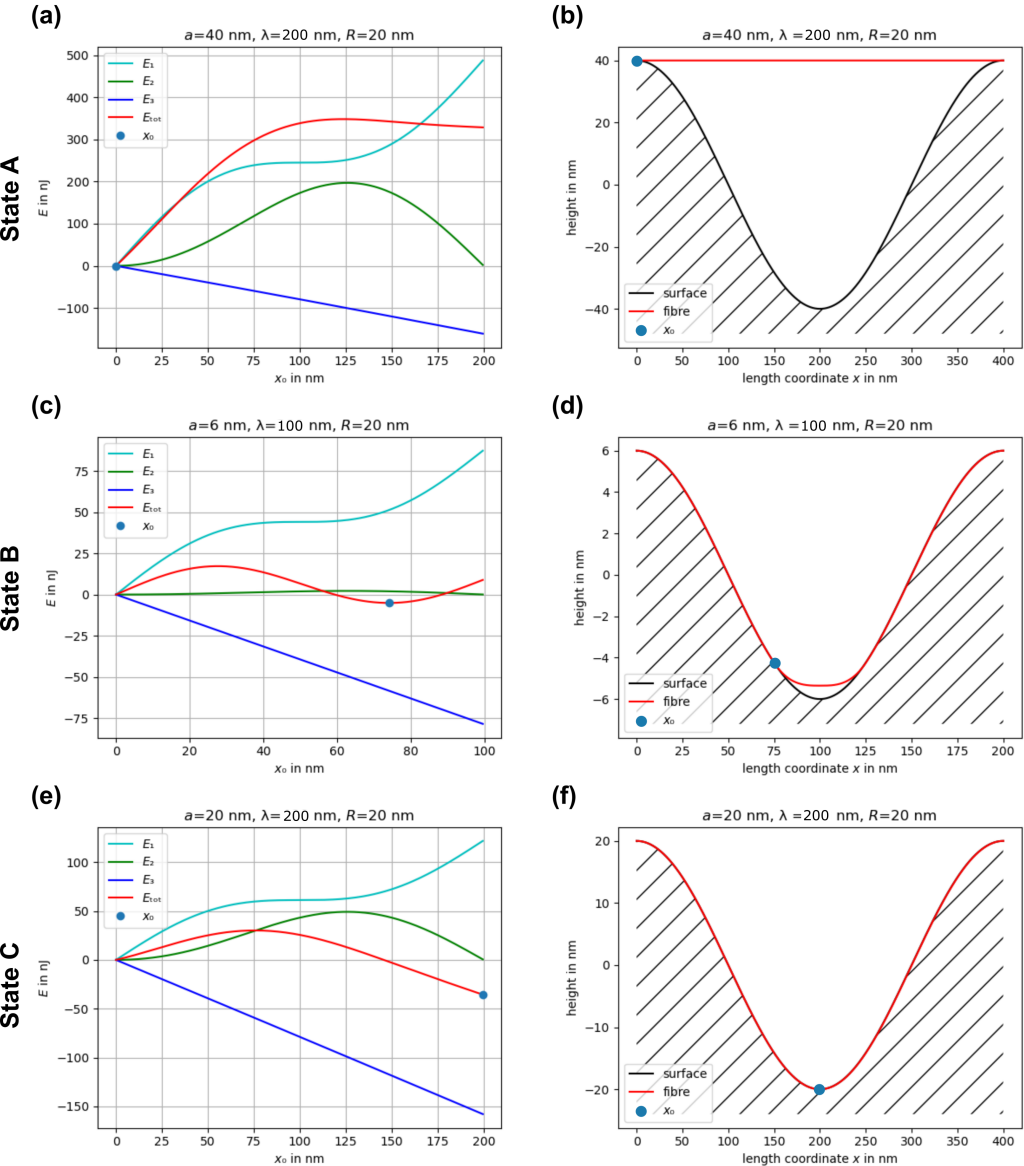
The three possible states: A (a, b), B (c, d), and C (e, f). (a, c, e) show the total energies *E*_tot_ as function of the parameter *x*_0_. (b, d, f) show the corresponding energetically most favorable shape of the fiber adaption to the surface. The blue full circles mark *x*_0_, at which *E*_tot_ is minimal and, thus, the point where the fiber detaches from the surface. The elastic modulus is assumed to be *E* = 80 MPa and *S* = 0 N. *a* denotes the amplitude, λ denotes the half of the period of the sinusoidal surface cross section and *R* is the fiber radius.

The energies and the corresponding shape of the fiber adaption to the surface of these three possible states, with an assumed elastic modulus *E* = 80 MPa (a typical value for spider silk [31]) and *S* = 0 N, are shown in Figure 3. Figure 3a,b shows state A, Figure 3c,d shows state B, and Figure 3e,f shows state C.

State B only exists in a very narrow parameter window and, thus, can be neglected. We were able to find a solution for the transition from state C, in the following also called the adhesive state, into an anti-adhesive state A, in dependence on only a few parameters and some constants such as the Hamaker constant and the elastic modulus of the fiber. These free parameters are the amplitude *a* and the spatial period Λ = 2λ of the sinusoidal surface, as well as the bending stiffness and the radius of the fiber in the case of *S* = 0.

While finding the minimum of the total energy is cumbersome when trying to find an analytical solution, the slope of the total energy can be calculated as


[25] $${\frac{\partial \left(E_{1}+E_{2}+E_{3}\right)}{\partial x_{0}}|}_{x_{0}=0}=\frac{\pi^{4}\cdot EJ\cdot a^{2}}{2\lambda^{4}}-\mu .$$

Thus, if


**Figure 4:**
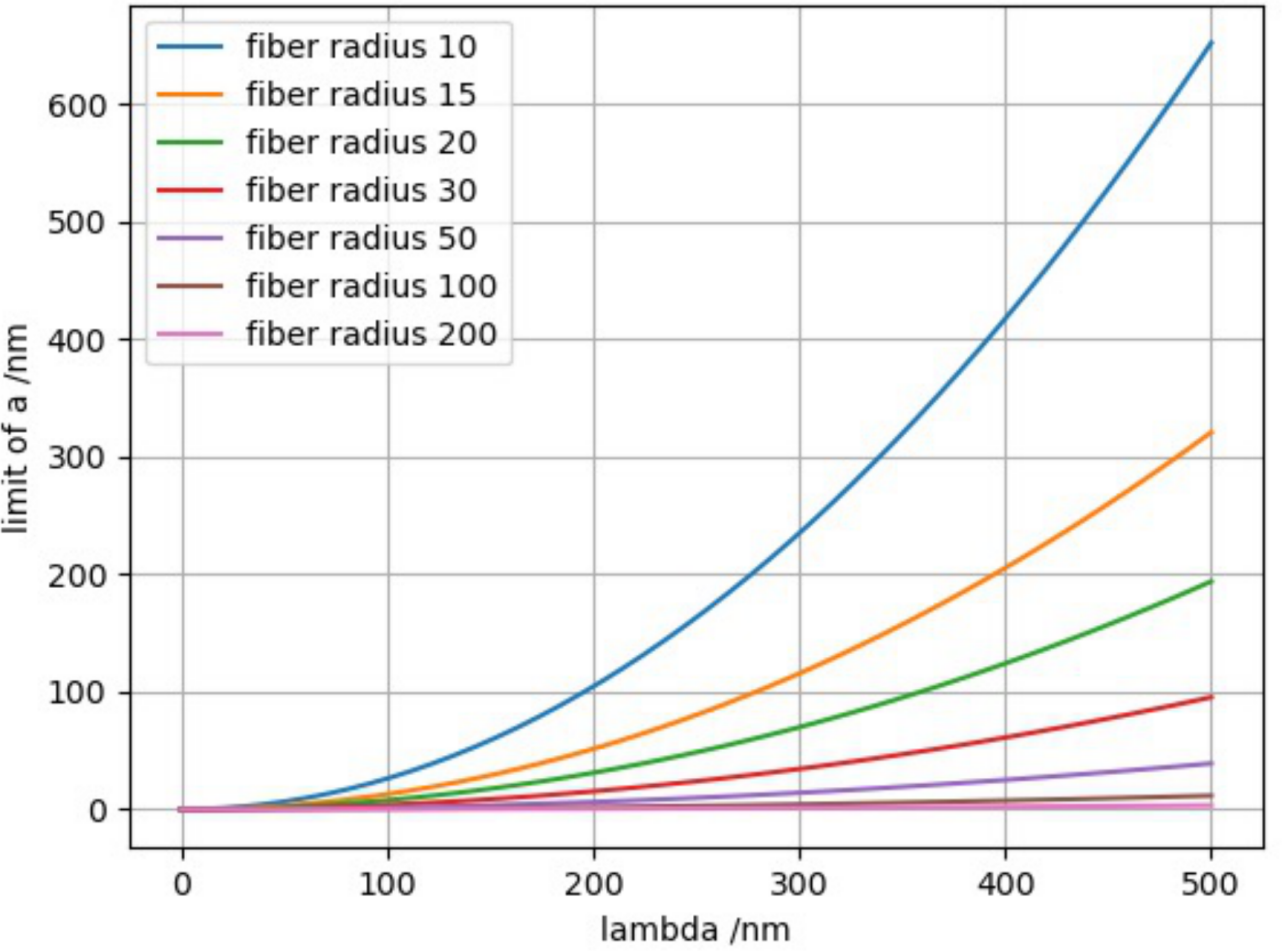
Transition from adhesive to anti-adhesive state for varying fiber radii ranging between 10 and 200 nm. The individual curves represent a lower limit of the surface modulation amplitude *a*, that is, the surface is adhesive for nanofibers through van der Waals forces only for values below the corresponding curves.

[26] $$a\leq \sqrt{\frac{2\lambda^{4}\cdot \mu }{\pi^{4}\cdot EJ}},$$

the fiber will adhere well as the energy directly decreases from *x*_0_ = 0. The results are shown in Figure 4 for *E* = 80 MPa, a Hamaker constant *A*_H_ = 7.5·10^−2^, and for different fiber radii *R* (in nm).

**Figure 5:**
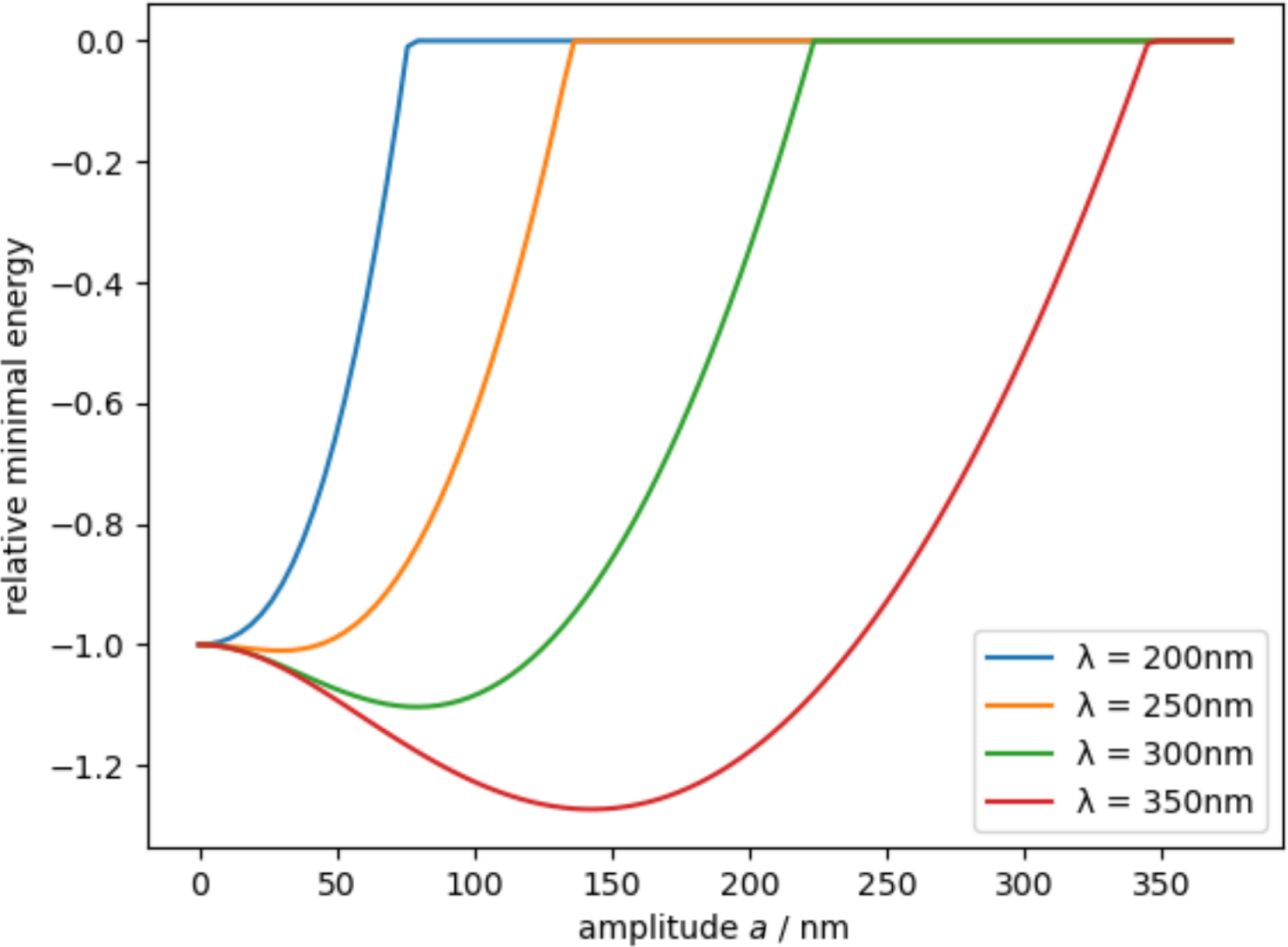
Relative total energy for different characteristic lengths λ for a fiber radius of *R* = 15 nm (the ordinate is normalized to a flat surface). One can see that the total energy initially decreases with increasing amplitude *a* with regard to a flat surface and, therefore, the adhesion increases if the amplitude *a* is too low.

Another interesting phenomenon that can be described with this theory is that the total energy *E*_tot_ initially decreases with increasing amplitude *a* with regard to a flat surface. With increasing λ the effect becomes stronger. This means, that if the aspect ratio between amplitude *a* and λ is too low, the surface becomes even more adhesive for nanofibers than flat surfaces. Figure 5 shows the above-described effect for a fiber radius *R* = 15 nm and different characteristic lengths λ.

### Design and test of LIPSS-covered metal surfaces that are anti-adhesive for electrospun PA-6 fibers

**Figure 6:**
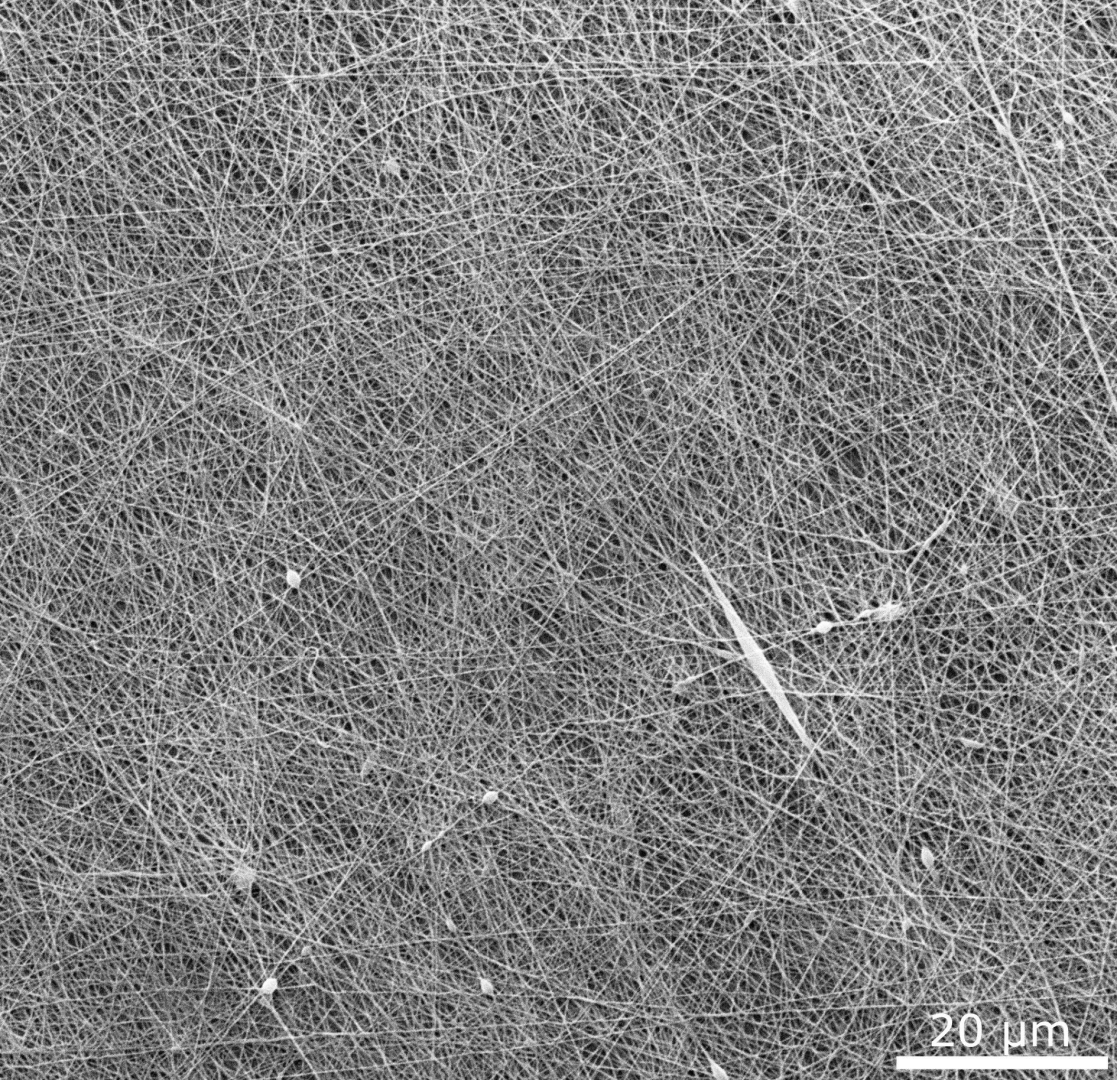
Scanning electron micrograph of electrospun nanofibers. One can see the random orientation of the individual fibers forming a kind of mesh.

In order to test the theory derived above, electrospinning of polyamide 6 (PA-6) in a laboratory setup onto structured metal samples (aluminum alloy, steel, and titanium alloy) was performed and the peel-off forces were determined using a custom-made peel-test device. PA-6 has an elastic modulus *E* of 0.6 up to 2.5 GPa, dependent on the treatment and the environmental conditions [32]. With our custom-made electrospinning setup, the typical diameters of the fibers are above 140 nm. Thus, from Equation 26, we can follow that adhesion is impaired if the modulation depth (double the amplitude) is approximately 2*a* > 250 nm when assuming *E* = 1 GPa, *R* = 70 nm, and an average in-surface-plane inclination angle of the fiber of 45° with respect to the ripple ridge direction. The angle is used to take statistically into account that the fibers will orient randomly on the surface, that is, the fibers form a kind of mesh on the surface ripples, as can be seen in Figure 6. Thus, not all fibers will orient perpendicular to the surface ripples. During electrospinning, the first contact with the surface takes place in form of a single, thin nanofiber. Therefore, the theory presented above, where a single, thin fiber is assumed is still valid in this case. The modulation depth that typically can be achieved for LIPSS (LSFL type [33]) on metals is up to 400 nm. Thus, an antiadhesive effect can be assumed.

**Figure 7:**
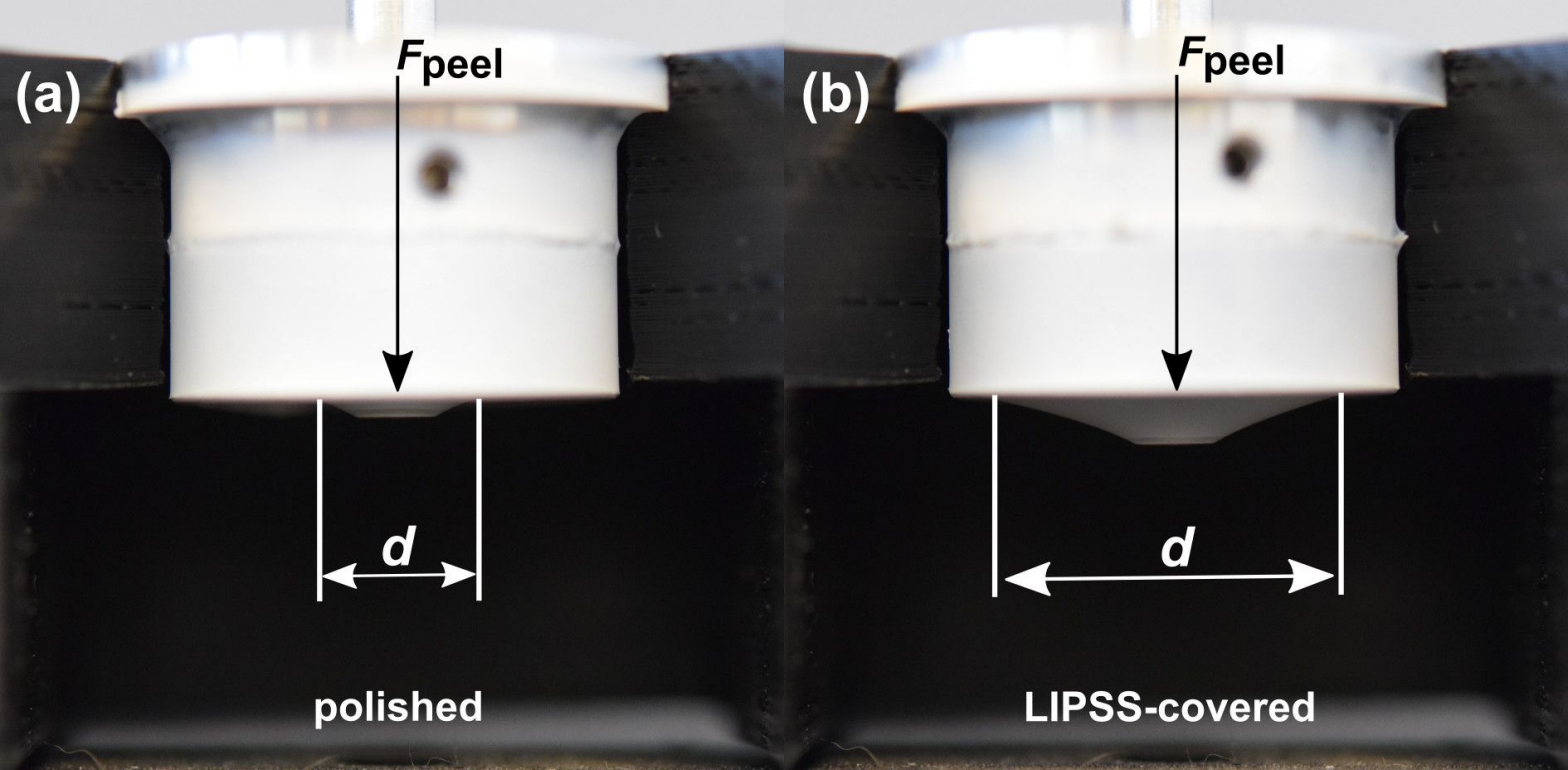
Peel-off force measurement of polished (a) and LIPSS-covered (b) steel samples. The applied weights and hence the normal forces are equal in panels (a) and (b). One can see that the cone diameter *d*, at equal applied forces, is larger on the LIPSS-covered sample than on the polished sample, which indicates that the peel-off force per unit length is smaller for the laser-structured samples.

The peel-off force, that is, the force per unit length of the peeling edge necessary to separate the nonwoven from the surface, was measured for the LIPSS-covered samples, for polished surfaces, that is, flat samples as control, and for randomly rough surfaces produced by means of facing and grinding (sandpaper with grit size 80 and P240). Scanning electron micrographs of all samples can be found in Figure S1 in Supporting Information File 1. In Figure 7, the peel-off force measurement is exemplified for the polished (Figure 7a) and LIPSS-covered (Figure 7b) steel samples. The applied weights and, hence, the normal forces are equal in Figure 7a and Figure 7b. The cone diameter *d* of the nanofiber layer on the LIPSS-covered sample is larger than that on the polished sample, which indicates that the peel-off force for the laser-structured samples is lower than for the polished samples.

**Figure 8:**
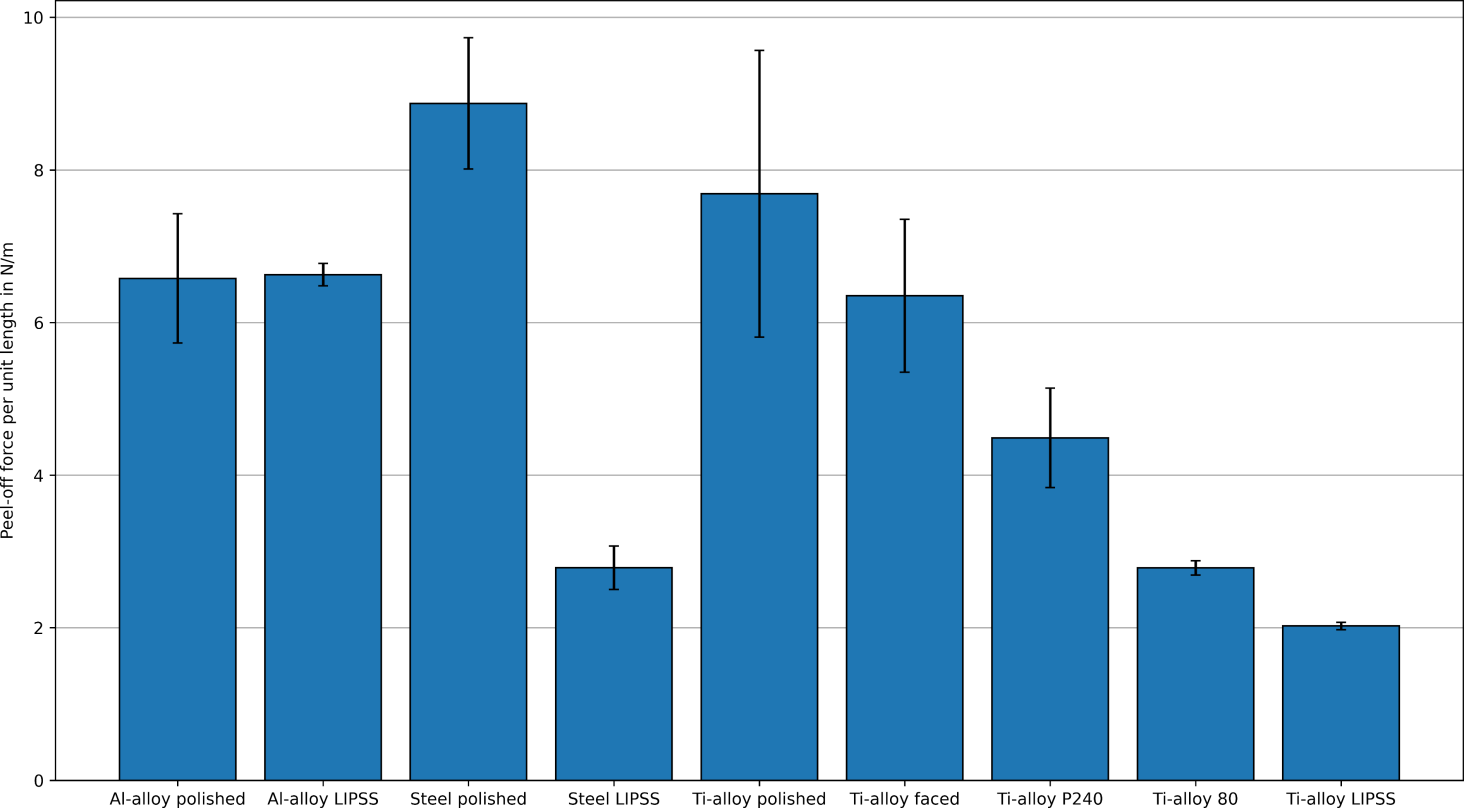
Peel-off force per unit length measurement results from Table 1 and Table 2 (mean values) visualized as bar plot. The error bars denote the standard deviations. Except for the Al alloy samples, the peel-off force for the LIPSS-covered samples is significantly lower than for the polished control samples and for the samples with randomly rough surface.

Nine different sample classes, that is, aluminium (Al) alloy polished, Al alloy LIPSS-covered, steel polished, steel LIPSS-covered, titanium (Ti) alloy polished, Ti alloy LIPSS-covered, Ti alloy faced, Ti alloy P240 (ground with P240 grit size sandpaper), and Ti alloy 80 (ground with 80 grit size sandpaper), were investigated as shown in Figure 7. For every sample class, *n* = 5 different measurements with different weights were performed and the mean values and the standard deviation were calculated for every sample class. Between the measurements, the samples were cleaned with 80% ethanol. The measured values of the peel-off forces per unit length needed for the LIPSS-covered and polished samples are listed in Table 1, the measured values of the randomly rough surfaces are listed in Table 2. All measurement results are also graphically shown as bar plots in Figure 8.

**Table 1:** Peel-off force per unit length measurement results for the polished and LIPSS-covered samples.

Meas. no.	Al alloy polished (N/m)	Al alloy LIPSS (N/m)	steel polished (N/m)	steel LIPSS- covered (N/m)	Ti alloy polished (N/m)	Ti alloy LIPSS (N/m)

1	5.57	6.40	7.32	2.67	5.87	2.06
2	6.12	6.61	8.63	2.54	6.43	2.03
3	6.03	6.56	9.15	2.64	6.26	2.06
4	7.46	6.80	9.70	2.74	9.40	1.93
5	7.71	6.77	9.56	3.34	10.48	2.04

mean value	6.58	6.63	8.87	2.79	7.69	2.02
standard deviation	0.85	0.15	0.86	0.28	1.88	0.05

**Table 2:** Peel-off force per unit length measurement results for randomly rough samples.

Meas. no.	Ti alloy faced (N/m)	Ti alloy P240 (N/m)	Ti alloy 80 (N/m)

1	5.31	3.99	2.92
2	5.36	4.04	2.63
3	6.02	4.06	2.82
4	7.39	4.65	2.79
5	7.68	5.70	2.76

mean value	6.35	4.49	2.78
standard deviation	1.00	0.65	0.09

According to Table 1, the mean peel-off forces per unit length were 2.02 N/m for the LIPSS-covered Ti alloy and 2.79 N/m for the LIPSS-covered steel samples and, thus, significant lower than for the corresponding polished surfaces (7.69 N/m for Ti alloy and 8.87 N/m for steel). Thus, for both metals (Ti alloy and steel), the LIPSS reduce the adhesion forces by approximately 70–75% compared to the polished surfaces. For the aluminium alloy sample, the presence of LIPSS had no significant influence on the peel-off force.

Furthermore, according to Table 2, the faced Ti alloy sample shows no significant difference in peel-off force compared to the polished control sample. The Ti samples ground with sandpaper showed a peel-off force reduced by approximately 40% (P240 grit size) and 65% (80 grit size), respectively. The investigated randomly rough surfaces did not reach the low peel-off forces for the LIPSS-covered samples. To prove the statistical significance of the measurement results, a statistical analysis was performed using Microsoft Excel. Details are presented in Table S2 in Supporting Information File 1.

To investigate the effects of the surface texture on the measured peel-off force in more detail, the surfaces of the Al alloy and the Ti alloy samples were investigated by means of atomic force microscopy (AFM). The arithmetic average roughness (*Ra*), the quadratic average roughness (*Rq*) and the maximum height (*Ry*) were measured; the results are shown in Table 3.

**Table 3:** Arithmetic average roughness (Ra), quadratic average roughness (Rq) and maximum height (Ry) of the samples measured by means of AFM.

Sample	*Ra* (nm)	*Rq* (nm)	*Ry* (nm)

Al alloy LIPSS-covered	19.7	25.0	170.0
Ti alloy LIPSS-covered	26.0	31.0	370.0
Ti alloy polished	3.3	4.6	19.7
Ti alloy faced	105.6	134.5	873.7
Ti alloy P240	86.0	108.0	909.0
Ti alloy 80	115.3	166.7	1450.0

The results of the AFM measurements revealed that the LIPSS covering the Al alloy surface are rather flat. *Ry*, which represents basically double the amplitude *a* of the LIPSS, is only 170 nm and, therefore, too low to reach the antiadhesive state according to the above theory (2*a* > 250 nm). This explains why the peel-off force measurement did not show a significant difference for the polished control and the LIPSS-covered Al alloy sample. Instead, the mean value is slightly higher for the LIPSS-covered sample than for polished control sample, which correlates with the behavior described in Figure 5. However, this effect could not be significantly proven.

Furthermore, Table 3 shows that an increasing surface roughness does not necessarily entail a decreasing peel-off force in the case of nanofibers. For example, the *Ra* value for all randomly rough surfaces is much higher than that for the LIPSS-covered Ti alloy sample, but the measured peel-off force is much lower for the LIPSS-covered Ti alloy sample. In addition, despite the high roughness of the faced sample, the peel-off force is not significantly lower than that of the polished control sample. However, a trend that the peel-off force decreases with coarser grit size can be observed for the samples ground with sandpaper. Therefore, in case of nanofibers a simple very rough surface is not enough to significantly lower the peel-off force.

**Figure 9:**
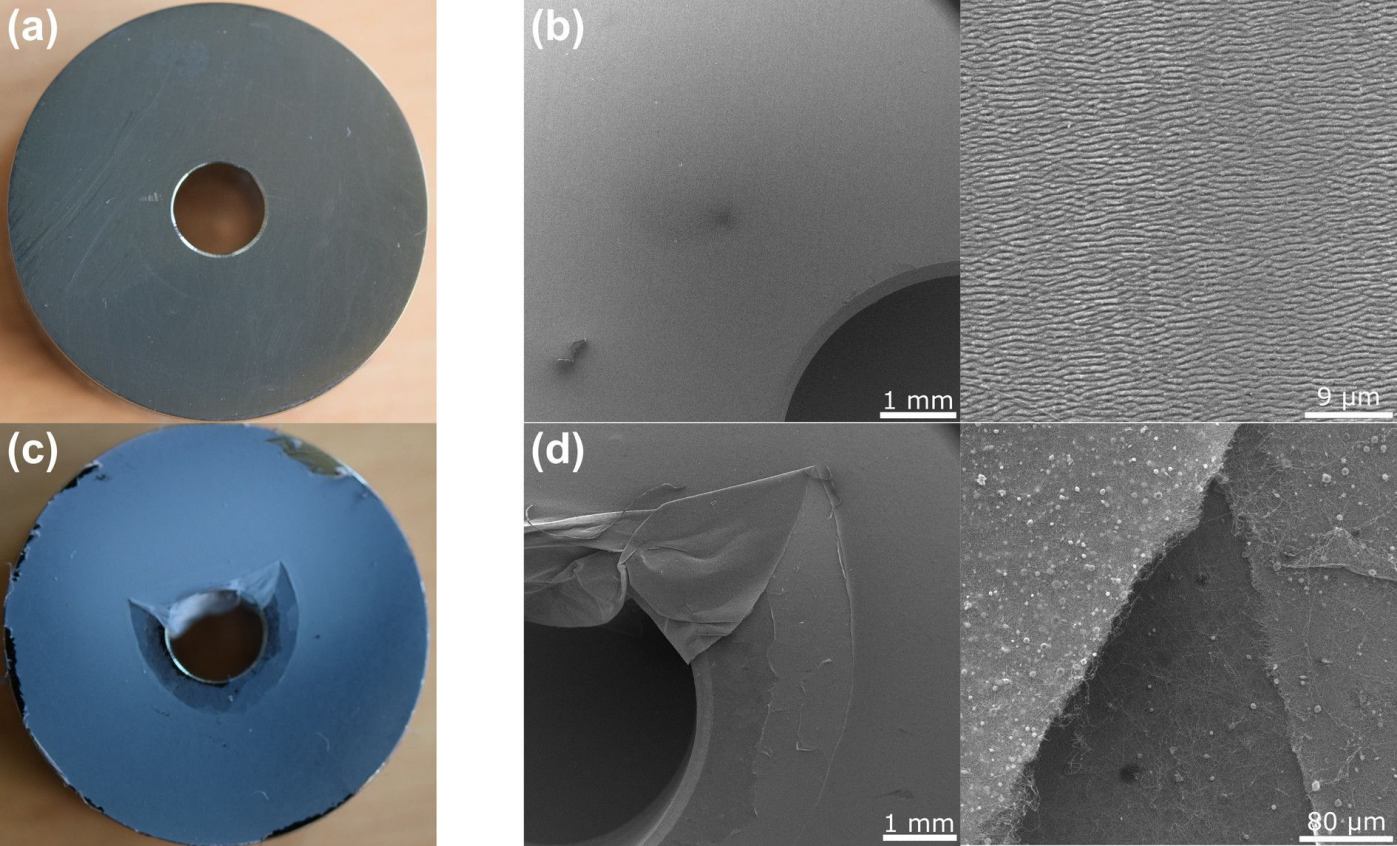
LIPSS-covered and polished titanium alloy surfaces after peel-off of an electrospun nonwoven. While the nanofibers could be removed entirely from the LIPSS (a, b), a film of nanofibers remains on the polished surface (c, d). This can be seen macroscopically (left) as well as in the SEM micrographs (right).

Additionally, due to the lower peel-off force on the laser-structured samples, no fibers can be found after testing on the laser-structured surfaces, whereas on the polished surfaces a layer of fibers remains (OM and SEM). This is shown exemplary for the titanium alloy samples in Figure 9. Thus, the force measured for peel-off from the polished surface is not the force needed to detach the fibers from the surface, but to tear a superficial fiber layer from the rest of the nonwoven.

## Conclusion

Mimicking the principle of nanostructures on the calamistrum of cribellate spiders, we were able to define an upscaled surface nanostructure with reduced adhesion force towards technical electrospun fibers. The biomimetic surface can be produced on metals by means of ultrashort pulse laser processing of self-organized laser-induced periodic surface structures, so-called LIPSS. For the technically relevant materials Ti alloy and steel, the presence of LIPSS reduced the peel-off forces by approximately 70–75% in both cases. Even more importantly, in contrast to the polished reference surface, no nanofibers remained at the LIPSS-covered surfaces. For the aluminium alloy samples, the presence of LIPSS had no significant influence on the peel-off force. This is due to the fact that the LIPSS on the aluminium alloy samples are rather flat and, therefore, do not have the required amplitude to reach the antiadhesive state. Furthermore, the measurements showed that a very rough surface does not necessarily entail a reduced peel-off force in case of cylindrical, thin nanofibers. Thus, one can conclude that the adhesion of electrospun nanofibers cannot be readily attributed to a simple roughness parameter, such as *Ra*. Rather, the correct aspect ratio of the sinusoidal surface structure as described in the above theory is important. Furthermore, as the experiments show, the sinusoidal structure based on the natural model allows us to adopt the structural dimensions to different fiber parameters, which is not possible with a random roughness of the surface.

It must be mentioned that the dimensions of the LIPSS are much larger than the dimensions of the fingerprint-like structure found on *Uloborus plumipes.* This is because the diameter of the fibers produced by *Uloborus plumipes* is much smaller than the mean diameter of the electrospun fibers. Therefore, the periodicity and the amplitude of the surface structure must be adjusted accordingly.

In this work, only one fiber type was investigated. However, in principle, these surface structures can be adapted to the requirements for different fiber types, that is, different diameters and elastic moduli, and, thus, different bending stiffness values within technical limits. Such non-adhesiveness is beneficial for tools or parts of tools used in the production of nanofibers. A reduced adhesion of nonwovens would not only help prevent residues of nonwoven on tools used in the production, but will also reduce the chance of tearing or generally damaging the nonwoven at separation from a target or a tool. As one can imagine, even small damages of the nonwoven during production could lead to severe problems in the application. Refinement and consequent application of the theoretical model described here might help to further optimize the surface structure and/or to find even better surface topographies to make nanofiber nonwovens easier to handle for a broader field of applications.

## Experimental

### Sample materials

Different metal alloys (grade-5 titanium alloy: Ti6Al4V, aluminium alloy: AlMg3) were purchased from Schumacher Titan GmbH (Solingen, Germany) and Gemmel Metalle (Berlin, Germany) as rods of 25 mm diameter. The rods were reduced to 24 mm diameter and cut into ca. 8 mm thick slabs. Circular slabs of hardened 100Cr6 steel (24 mm diameter, 8 mm thickness) were purchased from Optimol Instruments Prüftechnik GmbH (Munich, Germany). The top surfaces of the samples were mechanically polished resulting in a mirror-like surface finish with an average roughness *R*_a_ < 15 nm.

### Laser processing

The laser processing was performed using an ultrashort pulse laser (TruMicro 5050 femto edition, TRUMPF, Ditzingen, Germany) with a pulse duration of approx. 925 fs and a wavelength of 1030 nm, operated at 100 kHz pulse repetition rate. A galvanometer laser scanner (hurrySCAN II 14, SCANLAB GmbH, Puchheim, Germany), equipped with a f-theta lens of 160 mm focal length allowed us to scan the focused laser beam across the sample surface. The angle of incidence of the laser beam onto the sample surface was approx. 0°. The relative position of the sample to the scanner optics was adjusted to place the sample surface in the focal plane of the f-theta lens. At the sample surface, the focal diameter (1/*e*^2^) of the Gaussian laser beam was determined by the *D*^2^-method [34] as 2*w*_0_ = 35.5 μm. The samples were processed at optimized incident peak fluences of ϕ_0_ = 0.35 J/cm^2^ for Ti6Al4V, and 0.54 J/cm^2^ for 100Cr6 and AlMg3, while employing a meandering line-wise processing at a constant scan velocity of *v*_x_ = 700 mm/s and an inter-line separation of Δ = 5 µm. The linear laser beam polarization was kept parallel to the scan direction. Immediately after laser processing, the samples were cleaned for 5 min in acetone in an ultrasonic bath and stored in a desiccator.

**Figure 10:**
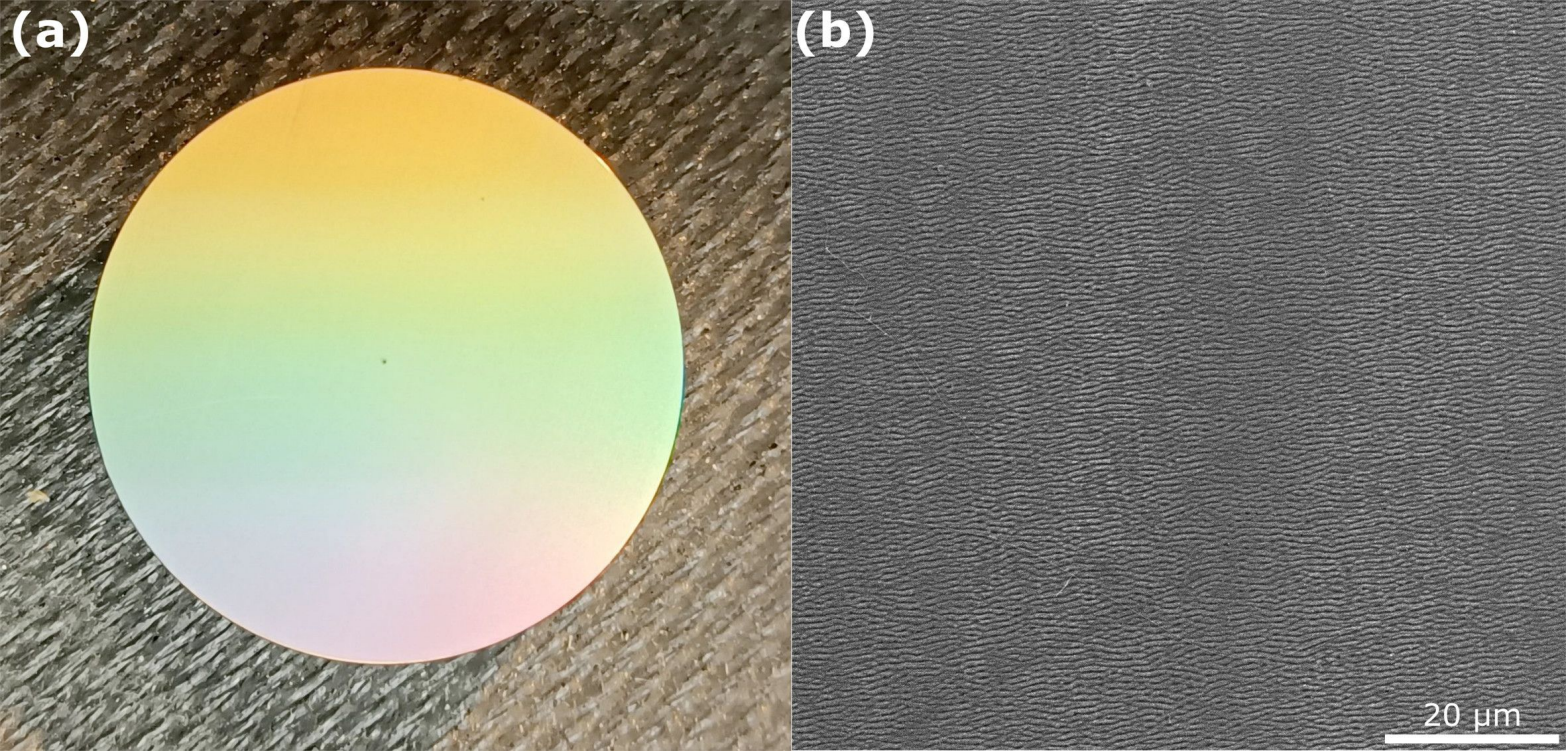
(a) Photography of a laser-structured titanium alloy sample after ultrafast laser processing. The colorful appearance arises from optical diffraction of the ambient natural light at the grating-like LIPSS-covered surface topography (structural colors). (b) Scanning electron micrograph of the LIPSS-covered titanium alloy surface.

Figure 10 indicates the presence of the LIPSS at the entire top surface of the laser-processed Ti6Al4V sample through structural color effects, that is, optical diffraction of the ambient light at the sub-micrometric grating-like LIPSS. High-resolution optical microscopy (OM) confirmed the presence of LSFL-LIPSS with average spatial periods Λ between 700 and 800 nm (data not shown here). A 5 mm borehole was mechanically drilled through the laser-processed disks prior to the adhesion measurements.

### Production of the randomly rough samples and measurement of the surface roughness

The Ti alloy samples with randomly rough surface were produced by means of facing and grinding with sandpaper. First, all Ti alloy samples were faced on a turning lathe. After facing, one sample was ground with sandpaper with grit size P240 and one sample was ground with sandpaper with grit size 80.

The surfaces of the polished, the LIPSS-covered, and the randomly rough Ti alloy samples, as well as the LIPSS-covered Al alloy sample were investigated by means of AFM on a Nanosurf Mobile S device (Nanosurf AG, Liestal, Switzerland). The investigated area had a size of 30 µm × 30 µm. The measurements were performed in contact mode with a Contr10 (Nanoworld, Neuchatel, Switzerland) pointprobe.

### Electrospinning

**Figure 11:**
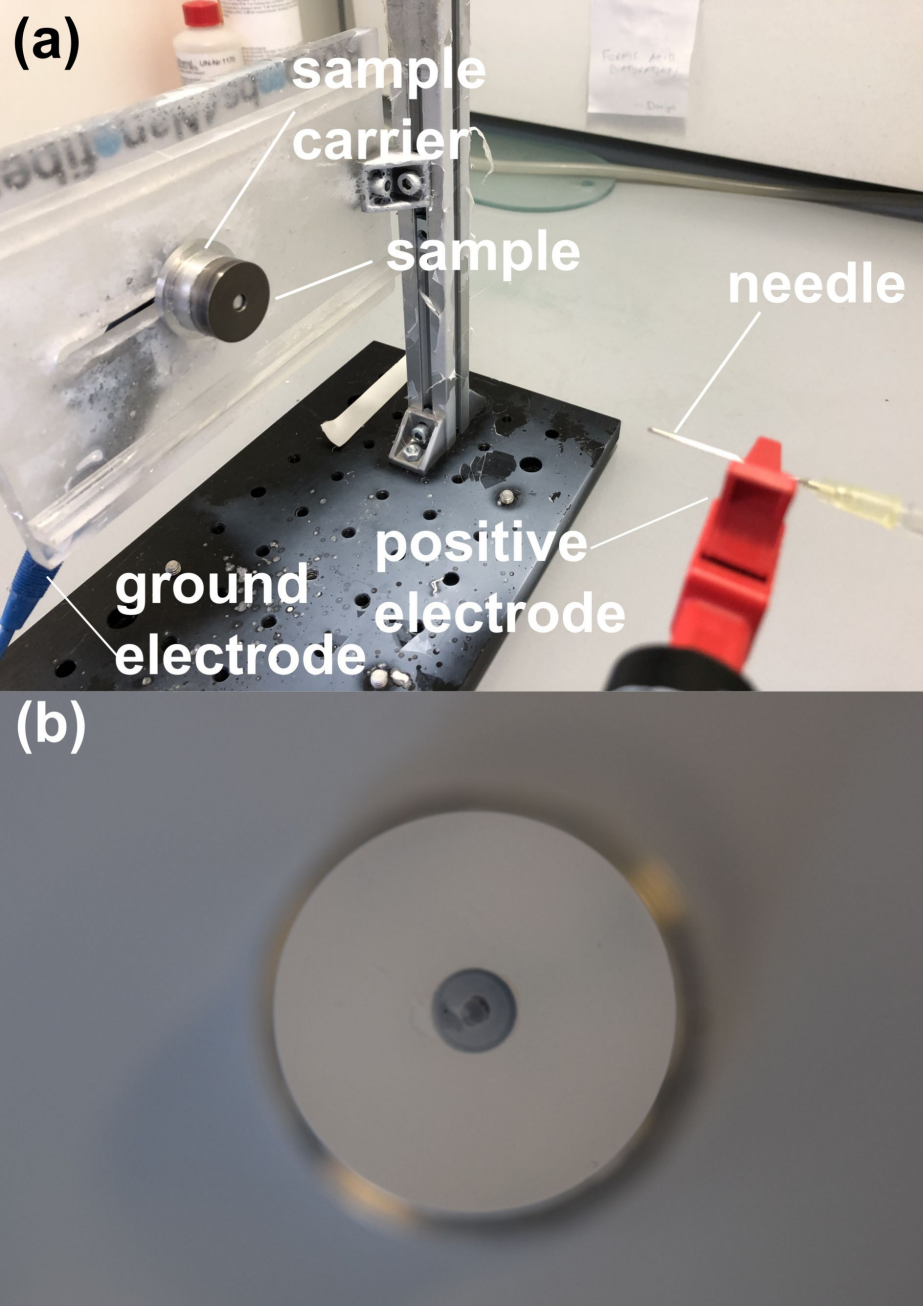
(a) Photography of the setup for the electrospinning process. (b) Top view of the spun sample after electrospinning, the surface is fully covered with a thin mesh layer of nanofibers (nonwoven).

Electrospinning was performed using a custom-made setup (Figure 11). The liquid PA-6 solution consisting of 6 g PA-6 polymer, 15 g formic acid, and 29 g acetic acid was mixed at 80 °C for about 90 min. The dope solution was delivered to the blunt needle tip (Sterican Standard Gr. 1 G20 x 1 ½’’, B. Braun SE, Melsungen, Germany) using a custom-made syringe pump at a flow rate between 0.2 to 0.3 mL/h (Figure 11a) equipped with a 1 mL plastic syringe (Omnifix-F, B. Braun SE, Melsungen, Germany). The sample fixed on an aluminium sample carrier was placed horizontally opposite to the needle at a distance of about 10 cm. The positive electrode of a high-voltage generator (HCP 35 – 35 000, FuG Elektronik GmbH, Schechen, Germany) was clamped onto the needle and the ground electrode was clamped onto the aluminium sample carrier. The corresponding voltage was set to 20 kV. The spinning process was performed until a uniform layer of nonwoven was achieved on the sample surface. Figure 11b shows a spun sample after the electrospinning process. The surface is fully covered with a thin mesh layer of nanofibers, that is, the nonwoven.

### Adhesion measurements

**Figure 12:**
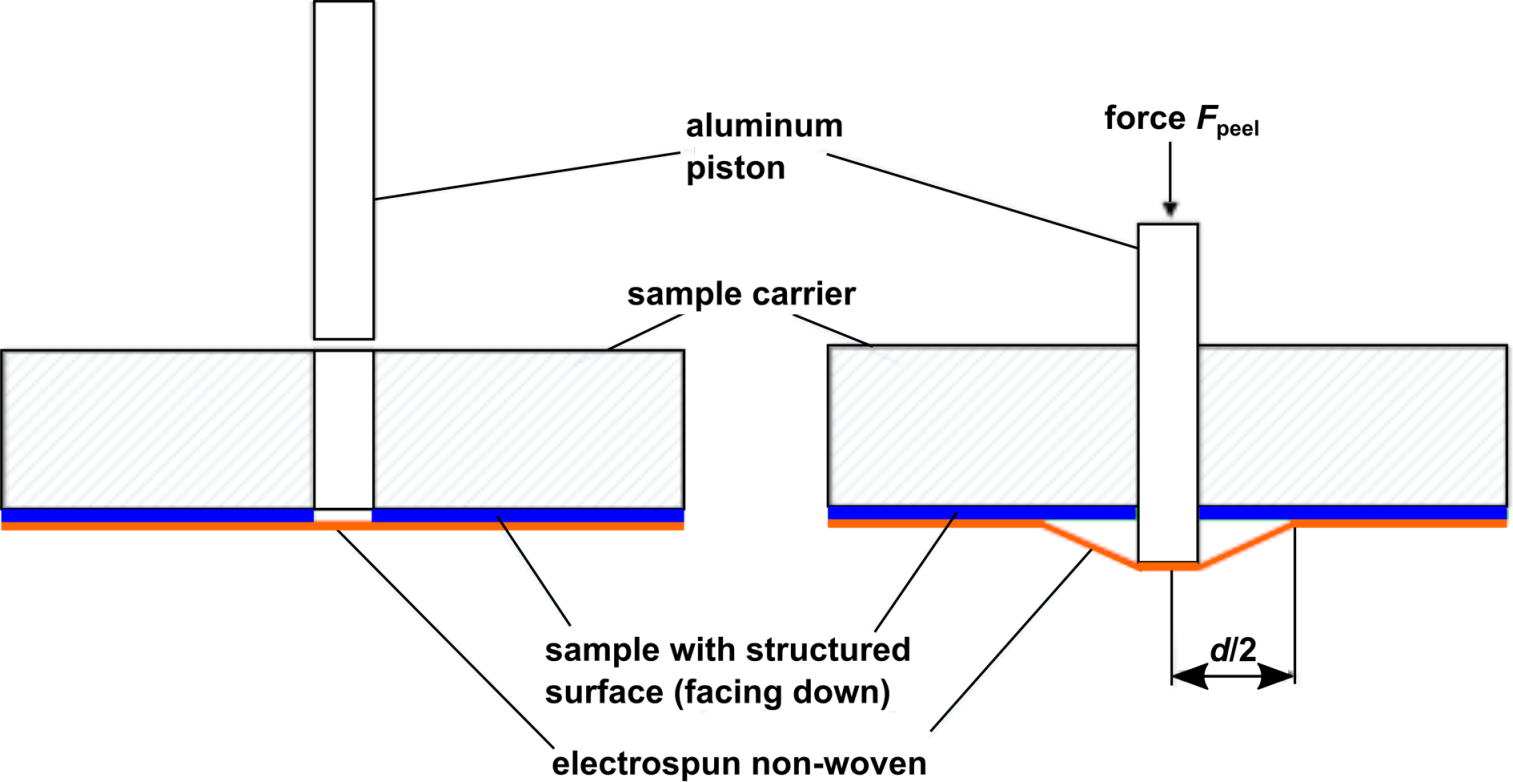
Measurement principle of the newly established peel-off test avoiding edge effects. Blue: sample with laser-structured surface; orange: electrospun nonwoven. Left: An aluminium piston is put into a 5 mm hole drilled into the sample carrier and the sample. Right: Defined forces *F*_peel_ are applied onto the aluminium piston by adding defined weights. When exceeding a critical threshold value, the peel-off forces separate the nonwoven from the sample surface, leading to the formation of a cone-like envelope with diameter *d*.

In order to quantify the adhesion of electrospun nonwoven, a new peel-off test had to be established. Existing peel-off tests are typically used for rather large samples; thus, the edge effects can be neglected. These effects become dominant when the width of the peeled homogeneous surface becomes small. In order to only peel the nonwoven from the interacting homogeneous surface and not from the sample edges (where unpredictable physical effects may take place) the adhesion test setup shown in Figure 12 was built.

The principle depicted in Figure 12 works as follows: The sample with a surface under investigation is mounted onto a sample carrier. Through this sample and the sample carrier a hole (diameter of 5 mm) is drilled. The hole is then filled with an aluminium piston that is fixed by a screw to form a straight surface. Then the nonwoven is spun onto the sample by means of electrospinning. After polymerization of the deposited nonwoven, the sample carrier is mounted into a holder and the fixation of the screw is removed. Then defined forces are applied onto the piston by placing weights on it. The nonwoven has only negligible bending stiffness macroscopically and, thus, the piston-induced force can only act longitudinal in the nonwoven. This force is split vectorial at the point of contact in a vertical peel off force and a local horizontal shear force. Due to the rotational symmetry the total horizontal forces cancel out. When exceeding a critical threshold value, the peel-off forces separates the nonwoven from the sample surface, leading to the formation of a cone-like envelope. Thus, the diameter *d* of the cone increases initially until, eventually, a force equilibrium occurs. This is due to the fact that the total peel-off force depends on the length *c* of the peeling edge. In our case this is


[27] $$F_{\text{peel}}=p\cdot c=p\cdot d\cdot \pi .$$

Here, *p* is the peel-off force per unit length, which is a measure for the adhesion of the nonwoven to the surface under investigation. Thus, by optically measuring the diameter *d* of the cone, the peel-off force per unit length can be determined from the known force *F*_peel_. The method was tested using siliconized paper and other materials, which were also tested by a commercially available peel-off meter. Both measurements were found to be in good agreement.

**Figure 13:**
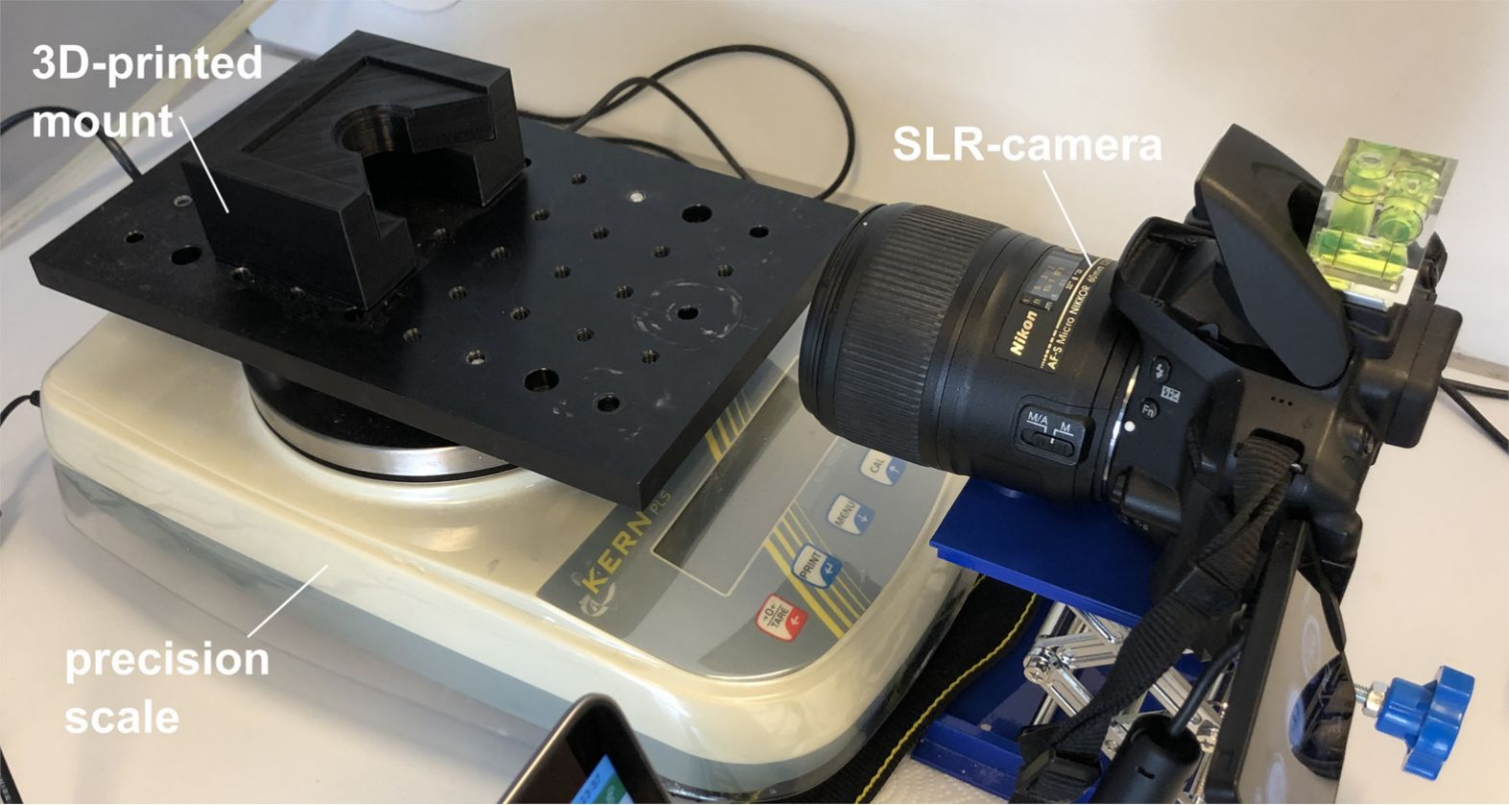
Setup used for the peel-off force measurements.

The measurement of the peel-off force was performed as follows: The investigated sample was fixed on the sample holder by means of double-sided tape and placed upside down in a custom-made 3D-printed mount. The whole setup was placed on a precision scale (Kern PLS 4200-2F, KERN & SOHN GmbH, Balingen-Frommen, Germany) and initially tared to zero. Then, successively different weights were applied, the measured weight was read from the display of the precision scale and a picture of the resulting cone was taken with an SLR-camera (Nikon D5300, Nikon Corporation, Tokio, Japan) equipped with a macro lens (AF-S Micro-NIKKOR 60 mm 1:2,8 ED, Nikon Corporation, Tokio, Japan). The resulting cone diameters *d* were then determined from the pictures using the free software Inkscape (V. 1.2.1, Inkscape Community). The peel-off was then calculated according to Equation 27 using Microsoft Excel for Mac (V 16.62, Microsoft, Washington, USA). The setup is depicted in Figure 13.

### Measurement of the calamistral nanoripples

The species used in the experiments is not an endangered or protected species. Special permits were not required. All applicable international, national, and institutional guidelines for the care and use of animals were followed.

*Amaurobius similis* (Blackwall, 1861) and *Uloborus plumipes* (Lucas, 1846) were captured in Aachen, Germany, and kept in the laboratory before being frozen at −20 °C. *Jamberoo johnnoblei* (Gray & Smith, 2008) was captured in the wild at Mt. Wilson (Australia) and kept in the laboratory before preserving the species in 70% ethanol. Specimen of *Hickmania troglodytes* (Higgins & Petterd, 1883) preserved in 70% ethanol were kindly provided by the Australian Museum (Sydney, Australia). The fourth leg, bearing the calamistrum, was removed from the ethanol-preserved specimens, dried in an ascending ethanol series, which was finally substituted by hexamethyldisilazane. After evaporation, the specimens were gold-sputtered (Hummer Technics Inc., Alexandria, USA; 7–10 mA, 5 min) and studied with the SEM (525 M, Philips AG, Amsterdam, Netherlands). Legs of *U. plumipes* were air-dried and otherwise prepared the same way for SEM analysis. Measurements were performed with ImageJ’s FIJI software [35].

Legs of *A. similis* were air-dried and gold-sputtered (S150B, Edwards). The metatarsi were examined using a focused ion beam scanning electron microscope (FIB-SEM) tomography (Strata 400 STEM, FEI Company, Oregon, USA) at the Central Facility for Electron Microscopy at the RWTH Aachen University. Measurements were performed using the accompanying software (xT Microscope Control).

For all spiders, we measured the peak-to-peak amplitude of the nanoripples covering the calamistrum at the region of interest. In addition, FIB tomography was used to measure the depth of structure in *A. similis*.

## Supporting Information

File 1 Additional experimental data.
