# Supplementary material for: Laser-processed antiadhesive bionic combs for handling nanofibers inspired by nanostructures on the legs of cribellate spiders
Source: Beilstein J Nanotechnol. 2022 Nov 7;13:1268–83. doi: 10.3762/bjnano.13.105 (PMC9663977; doi:10.3762/bjnano.13.105)
Supplement: File 1 — Additional experimental data. [file Beilstein_J_Nanotechnol-13-1268-s001.pdf]

BEILSTEIN JOURNAL OF NANOTECHNOLOGY

Supporting Information

for

Laser-processed antiadhesive bionic combs for handling nanofibers inspired by nanostructures on the legs of cribellate spiders

Sebastian Lifka, Kristóf Harsányi, Erich Baumgartner, Lukas Pichler, Dariya Baiko, Karsten Wasmuth, Johannes Heitz, Marco Meyer, Anna-Christin Joel, Jörn Bonse and Werner Baumgartner

Beilstein J. Nanotechnol. **2022,** *13,* 1268–1283. doi:10.3762/bjnano.13.105

Additional experimental data

License and Terms: This is a supporting information file under the terms of the Creative Commons Attribution License (https://creativecommons.org/licenses/by/4.0). Please note that the reuse, redistribution and reproduction in particular requires that the author(s) and source are credited and that individual graphics may be subject to special legal provisions.
The license is subject to the *Beilstein Journal of Nanotechnology* terms and conditions: (https://www.beilstein-journals.org/bjnano/terms)

Table S1: Morphological data of the nanoripples found on the calamistrum of four distantly related cribellate spiders.

**Species**	**Periodicity (nm)**	**Height (nm)**
Amaurobius similis	222 ± 8<br>(n = 3)	210<br>(n = 1)
Hickmania troglodytes	205 ± 13<br>(n = 3)	
Jamberoo johnnoblei	233<br>(n = 1)	
Uloborus plumipes	216 ± 10<br>(n = 3)	200[a]

[a]Data taken from [1].

Table S2: Statistical analysis of the presented measurement results (Two-tail *t*-test assuming equal variances, confidence level 95%).

**Sample 1**	**Sample 2**	**P value**	**Statistically significant**
Al alloy polished	Al alloy LIPSS	0.91	No
Steel polished	Steel LIPSS	8.85e−7	Yes
Ti alloy polished	Ti alloy LIPSS	3.14e−4	Yes
Ti alloy faced	Ti alloy LIPSS	2.49e−5	Yes
Ti alloy 240	Ti alloy LIPSS	6.66e−5	Yes
Ti alloy 80	Ti alloy LIPSS	4.76e−7	Yes
Ti alloy faced	Ti alloy polished	0.25	No
Ti alloy 240	Ti alloy polished	0.01	Yes
Ti alloy 80	Ti alloy polished	8.09e−4	Yes

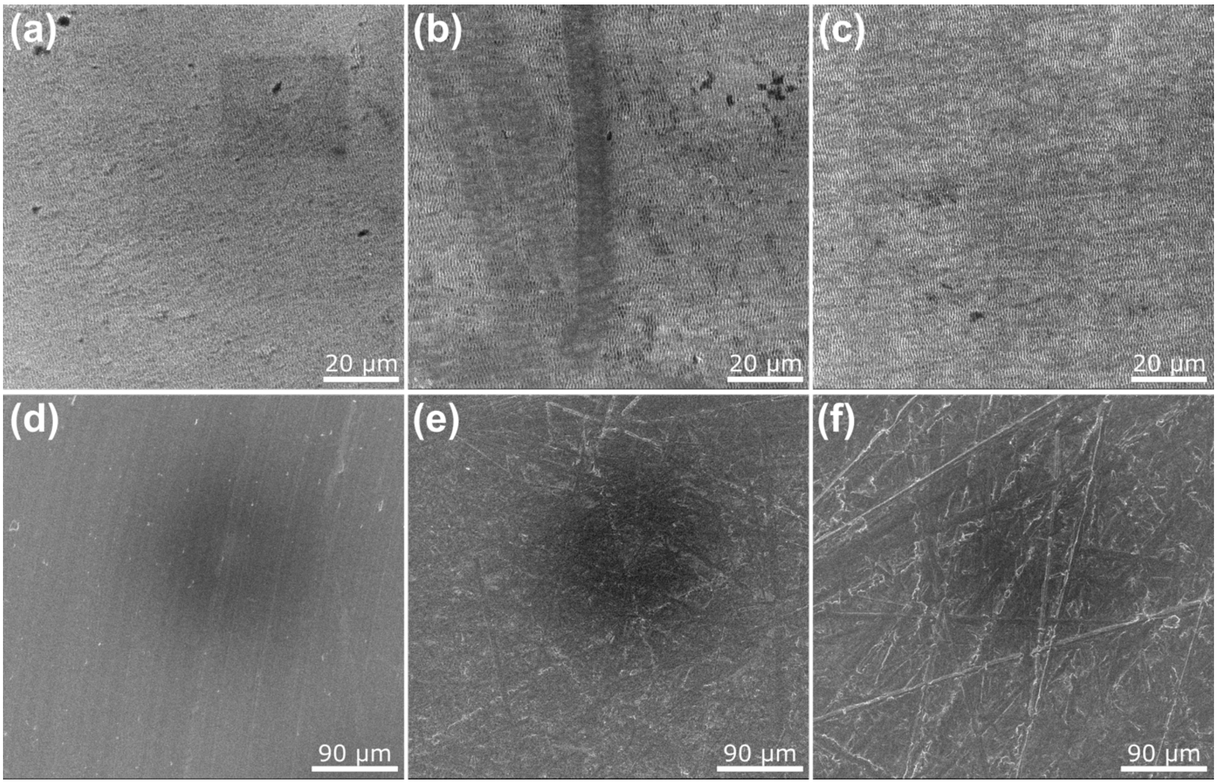

Figure S1: Scanning electron micrographs of the surface of the investigated samples. (a) Al alloy LIPSS-covered; (b) steel LIPSS-covered; (c) Ti alloy LIPSS-covered; (d) Ti alloy faced; (e) Ti alloy 240; (f) Ti alloy 80.

References

1. Joel, A.-C.; Meyer, M.; Heitz, J.; Heiss, A.; Park, D.; Adamova, H.; Baumgartner, W. *ACS Appl. Nano Mater.* **2020**, *3*, 3395–3401. doi.org/10.1021/acsanm.0c00130
